# An open-source spectral measurement platform for plant reflectance and material identification

**DOI:** 10.1016/j.ohx.2026.e00768

**Published:** 2026-04-01

**Authors:** M. Mejia-Herrera, J.S. Botero-Valencia, J.F. Vargas-Bonilla, J.M. Pearce

**Affiliations:** a Grupo de Sistemas de Control y Robótica (GSCR), Instituto Tecnológico Metropolitano, Medellín, Colombia; b Sistemas Embebidos e Inteligencia Computacional (SISTEMIC), Universidad de Antioquia, Medellín, Colombia; c Department of Electrical & Computer Engineering and Ivey Business School, Western University, London, ON, Canada

**Keywords:** Spectral sensing, Reflectance measurement, Open-source hardware, Vegetation analysis, Material identification

## Abstract

Spectral sensing plays a crucial role in agriculture, environmental monitoring, and material characterization, providing non-destructive insights into chemical composition and physiological status of plants. Commercial spectrometers, however, are often expensive and lack modularity, which limits their adoption in educational, research and field applications. The developed open-source spectral measurement platform addresses this gap by offering a low-cost, customizable solution for reflectance analysis in plant leaves and diverse materials. The system integrates a Hamamatsu C12880MA miniature spectrometer with dual illumination: a white LED and a 3 mm incandescent bulb, covering 340–850 nm. Control and acquisition are managed by a Teensy 3.2 microcontroller through a Python interface that enables calibration and data storage. The modular enclosure, fabricated via RepRap-class fused filament-based 3D printing and PVC components, ensures flexibility and reproducibility. Validation tests demonstrated accurate wavelength alignment when exposed to laser sources, with peaks detected at 407 nm and 663 nm, and mean squared errors of 0.0131 and 0.0208, respectively. Ambient light comparisons with a commercial OSHP spectrometer yielded an MSE of 0.0105 (≈10%), indicating strong agreement. Reflectance measurements using a ColorChecker Classic confirmed consistency in 340–850 nm, validating the suitability of the device for plant reflectance analysis and material identification in both scientific research and educational settings.

## Graphical abstract

**Figure:**
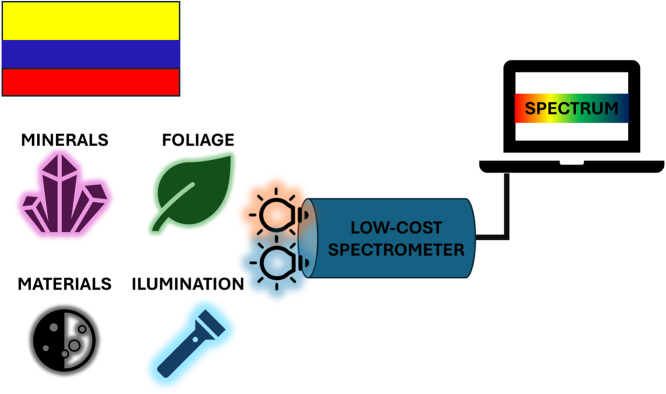

## Specifications table

**Table:** 

Hardware name	*Low cost radiance/Reflectance spectrometer*

Subject area	• *Engineering and material science* • *Environmental, planetary and agricultural sciences* • *Educational tools and open source alternatives to existing infrastructure*

Hardware type	• *Imaging tools* • *Measuring physical properties and in-lab sensors* • *Biological sample handling and preparation* • *Field measurements and sensors* • *Electrical engineering and computer science* • *Mechanical engineering and materials science*

Closest commercial analog	*Reflectance spectrometer OSH350s $1600*
Open source license	Creative Commons Attribution-ShareAlike license
Cost of hardware	*$442.89 USD*
Source file repository	*OSF — Design and Implementation of a Low Cost Spectrometer*
OSHWA certification UID	OSHWA Certification, OSHWA UID **CO000001**

## 1 Hardware in context

Optical spectrometry is one of the most widely used techniques for material characterization in various scientific disciplines. It involves acquiring a spectral representation (known as a spectral signature) derived from the electromagnetic spectrum emitted, transmitted, or reflected by an object or sample. This signature enables the identification or differentiation of materials, the assessment of the vegetation status [1], [2], [3], [4], the evaluation of the water quality [5], [6], [7], [8], [9], soil characterization [10], [11], [12], [13], [14], [15] and the determination of food quality [16], [17], among other applications [18], [19], [20], [21], [22], [23], [24], including the impact of albedo on the performance of solar photovoltaics [25], [26] and the spectral impacts of lighting on food crops [27], [28], [29].

Reflectance spectrometry places the spectral sensor and the light source within the same device, both directed toward the target. The illumination source projects light onto the object, which is then reflected toward the sensor, where it is diffracted to capture its spectral signature. Reflectance spectrometry is, therefore, an optical technique that allows for determining the proportion of radiation from a known source that is reflected by a target object. This type of spectrometry has recently gained interest in agriculture due to its potential to support irrigation and fertilization strategies, aiming to reduce the consumption of water and nutrients [1], [2], [3], [17], [30], [31], [32]. Although it is a powerful technique that has been in use for several years, it remains costly; Commercial systems can range from $2000 USD and more, depending on their specifications and resolution [33], [34], [35], [36], [37], [38]. This situation hinders the adoption of these technologies in developing countries, many of which are economically dependent on agricultural production and exports [39]. For this reason, agricultural communities or agricultural entities often monitor crops using soil chemical analysis or destructive sampling methods to determine the chemical composition of plants. These techniques are also often expensive and time-consuming, making real-time, in-field decision-making more difficult [40].

To overcome similar challenges in real-time monitoring and scientific equipment fabrication, it is well-known that open hardware principles reduce economic costs [41], [42], [43]. A recent review article found that in general the cost of scientific hardware could be cut by 87 percent as compared to proprietary commercial models with similar or lesser functionality using the open source approach and 94 percent when using digital manufacturing like 3D printing and open source electronics [44]. This reduction in open scientific hardware improves the accessibility of the equipment, particularly for under resourced labs [43].

The Clorofilo system [45] operates by using the ambient light sensor (ALS) integrated into a smartphone to measure light transmission through plant leaves. The device has an estimated total cost of approximately $65.26 and consists of a lighting circuit and a 3D-printed housing. The illumination system incorporates an LED band centered at 660 nm, a wavelength commonly used for chlorophyll-related optical measurements. The mechanical design is composed of eight smaller parts, allowing relatively straightforward assembly. The system, however, presents several limitations associated with its dependence on the smartphone’s ALS. Because the ALS specifications vary across smartphone models, the measurement precision is not consistent and may produce different responses depending on the device used. Additionally, many modern smartphones conceal the exact location of the ALS within the device enclosure, which complicates the alignment of the optical path between the external accessory and the sensor. This issue can hinder reliable sample acquisition and makes it difficult to ensure proper positioning of the device when clamped onto different smartphone models.

The system presented in [46] consists of a multispectral imaging platform operating across the visible to near-infrared (VIS–NIR) spectral bands. It integrates thermal imaging and environmental sensors to enable non-invasive agricultural analysis. By combining multiple sensing modalities with integrated data processing, the system monitors plants as dynamic systems that respond to environmental changes, providing measurements with high spatial resolution. The camera platform is designed for mobility and can be easily mounted on tripods or agricultural machinery, allowing extensive field coverage without requiring fixed sensor installations. Measurement precision is supported by the use of specialized narrow band-pass filters that isolate specific wavelengths, thereby improving the accuracy of the acquired spectral data. The system architecture incorporates a rotating filter wheel that holds multiple optical filters, which are sequentially positioned in front of the cameras to capture spectral information. This configuration enables each camera to acquire images across different spectral bands by selecting the appropriate filter for each acquisition cycle. Subsequently, the images obtained from the cameras are fused to generate a comprehensive spectral profile that supports non-invasive analysis in precision agriculture. This approach enhances the system’s ability to monitor plant responses to environmental conditions effectively. The implementation of a rotating filter wheel and multiple sensing modules, however, results in a considerable number of mechanical and electronic components, increasing the overall complexity of the system’s construction. Additionally, the presence of moving parts introduces potential maintenance challenges and may affect long-term reliability. The total cost of the system exceeds 2000 euros, which limits its accessibility and may hinder large-scale deployment in field conditions.

The system presented in [47] for measuring vegetation spectral signatures employs a FLAME-S VIS-NIR spectrometer capable of capturing spectral data within the 350–1000 nm range across 2049 spectral bands. This high spectral resolution enables detailed characterization of vegetation reflectance, which is particularly relevant for applications in precision agriculture and plant health monitoring. Two measurement protocols were implemented in the system. The first relies on natural sunlight as the illumination source, while the second utilizes an integrated HL-2000-LL light source from Ocean Insight, which provides a spectral range from 360 to 2400 nm. These alternative illumination strategies allow the system to operate under different environmental conditions and facilitate controlled measurements when natural illumination is not sufficient. Regarding the cost, the bill of materials reports a total expenditure of approximately €6947.86 when all components are considered, including mechanical elements such as screws and nuts, as well as the spectrometer itself. This relatively high cost may limit the accessibility and widespread adoption of the system, particularly in field-based applications. Several technical challenges have also been identified. The system exhibits elevated noise levels in specific spectral regions, particularly between 400–450 nm and 840–1000 nm, which can negatively affect measurement accuracy. Furthermore, proper system calibration is essential to improve the signal-to-noise ratio and ensure reliable spectral measurements, emphasizing the importance of precise integration and alignment of the hardware components.

The system presented in [48] describes a portable, low-cost IoT-based hyperspectral acquisition device designed for a wide range of indoor and outdoor applications. The platform integrates several key components, including a Particle Argon development board that enables wireless data transmission and onboard processing, AS7265x multispectral sensors, a rechargeable battery capable of providing up to 24 h of autonomy with a sampling interval of four seconds (extendable to approximately 10 days with longer acquisition intervals), and a Nano Power timer used to optimize energy consumption. The device enclosure is manufactured from acrylonitrile butadiene styrene (ABS) and complies with the IP67 protection standard, allowing reliable operation under outdoor environmental conditions. The system is designed to collect and analyze spectral data for applications in architectural engineering, intelligent lighting control, occupational health monitoring, and artwork preservation. Among its main advantages are the capability to support real-time decision-making, integration with intelligent energy monitoring systems, and the detection of potentially harmful infrared radiation. The system is capable of acquiring measurements across 18 spectral bands within the wavelength range from 410 nm to 940 nm. The system is primarily intended for radiation measurement however, and does not directly support reflectance measurements. Additionally, the architecture relies on the combination of three multispectral sensors to cover specific wavelength ranges. As a consequence, the spectral coverage is not continuous, resulting in regions where spectral information is absent. These gaps may limit the system’s ability to perform detailed spectral characterization compared with full-spectrum spectrometers.

Overall, existing systems demonstrate a trade-off between accessibility, spectral resolution, and system complexity. Low-cost solutions [45] provide affordable measurements but depend on variable consumer hardware, while advanced platforms [46], [47] offer higher precision at the expense of increased cost and mechanical complexity. Portable multispectral devices [48] improve field deployability but rely on discrete spectral bands that limit detailed spectral characterization. These limitations highlight the need for compact and low-cost spectroscopic systems capable of providing continuous spectral measurements while maintaining portability and ease of deployment.

Thus, this article presents an open-source optical reflectance spectrometer designed to operate within the spectral range of 340 nm to 850 nm. The system enables the acquisition of spectral signatures from target objects, providing researchers and technicians with an accessible tool for analyzing phenomena related to the electromagnetic spectrum. Although the system is designed as a low-cost alternative compared to commercial spectrometers, it incorporates the Hamamatsu C12880MA spectral sensor, which represents the most significant component of the total system cost. This sensor was selected due to its ability to provide continuous spectral measurements with high spectral resolution across the visible and near-ultraviolet range, a capability that is typically unavailable in lower-cost multispectral alternatives based on discrete-band sensors such as the AS7265x family. While these alternatives offer lower cost, they capture only a limited number of spectral bands, which restricts detailed spectral characterization. Therefore, the C12880MA sensor was chosen to balance affordability with the need for continuous spectral acquisition.

Furthermore, the proposed system contributes to the democratization of spectroscopic technologies by providing a significantly more affordable platform compared to commercial instruments, whose cost often exceeds several thousand dollars. This accessibility facilitates the adoption of spectral analysis tools in research, educational environments, and applications such as agricultural monitoring and environmental studies, ultimately supporting improved resource management and strengthening food security. Finally, although the system is primarily designed as a reflectance spectrometer, it can also operate as a spectroradiometer for the analysis of optical phenomena and light sources.

## 2 Hardware description

The developed system is based on the Hamamatsu C12880MA spectral sensor, integrated with a white LED and a 3 mm incandescent bulb. System control is handled by a Teensy 3.2 board (hereafter, Teensy), which is based on the ARM Cortex-M4 architecture. The device includes a backlit power button and a USB Type-C female to micro USB Type-B panel-mounted connector, used for connection with the Teensy. The Hamamatsu C12880MA sensor enables the capture of spectral signatures across 288 bands, with a spectral resolution of 15 nm, covering the range from 340 nm to 850 nm, making it suitable for applications involving monitoring, analysis, and material identification. The system was designed in a modular fashion, allowing each component to be modified and adapted according to the needs of the end user, which facilitates the integration of different types of lighting. Additionally, the system enclosure is built from a 2-inch PVC pipe, allowing for simple and adaptable construction using commercially available fittings. This feature also enables resizing the system to incorporate additional hardware, such as batteries or complementary sensors.

The system is primarily designed for the acquisition of reflectance spectral signatures in foliar vegetation. For this purpose, it incorporates an integrated illumination module consisting of a white LED and a 3 mm incandescent bulb rated at 3 V and 60 mA, which provide complementary spectral coverage for vegetation studies. It also features a movable lid that can be opened or closed to secure the sample, creating a small capture chamber. This lid opens via a 90° rotation, allowing larger samples to be introduced and enabling the analysis of objects with varying geometries or illumination conditions.

In order to ensure the quality of the spectral measurements, several geometric and illumination considerations were taken into account during the design of the measurement chamber.

The C12880MA sensor has a numerical aperture of 0.22, which corresponds to an acceptance cone with an approximate angle of 25.4°. Considering that the sample is positioned at a distance of 20 mm from the sensor, an effective measurement diameter of approximately 9 mm is obtained, located in the central region of the measurement chamber. This geometry defines the specific region from which the reflected radiation is collected by the sensor.

To guarantee that this region receives sufficient illumination, the lighting system was configured to completely cover the area of study. The white LED used has an approximate opening angle of 120°, which at the operating distance corresponds to an illumination diameter of approximately 7 cm. The incandescent bulb, due to the geometry of its filament and the absence of collimating optics, emits radiation in a nearly omnidirectional pattern, resulting in a coverage area even larger than that of the LED. The overlap of both emission patterns ensures that the effective measurement diameter, corresponding to the 9 mm defined by the sensor’s acceptance cone, remains fully contained within the illuminated area, guaranteeing that the analyzed region receives sufficient and homogeneous irradiance.

Since the acquisitions are performed with the chamber completely closed, and in order to ensure the quality and reproducibility of the measurements, it is recommended to use samples with dimensions similar to the internal area of the chamber. This consideration facilitates the correct alignment between the sample and the optical axis of the sensor, since any misalignment may generate significant errors in the recorded reflectance values.

Additionally, the samples are positioned orthogonally to the optical axis of the system in order to ensure reflectance measurements under normal incidence. This geometric configuration minimizes variations associated with changes in the angles of incidence and observation, which can modify the fraction of reflected radiation as a consequence of the bidirectional effects described by the bidirectional reflectance distribution function. Maintaining the surface perpendicular to the sensor establishes a controlled and reproducible angular condition, reducing unwanted specular contributions and improving comparability between measurements.

Furthermore, the approximate study diameter of 9 mm defines an integration area sufficiently large to average microscopic heterogeneities associated with the surface texture of the material. This spatial integration effect attenuates the influence of small local variations in roughness, microstructural orientation, or pigment distribution, favoring a more representative estimation of macroscopic reflectivity. Consequently, the system does not only record a point spectral response but rather a spatial average that increases the stability and repeatability of the measurements.

Mechanical parts of the system were manufactured using 3D printing via RepRap-class fused filament fabrication (FFF) [49], [50], with acrylonitrile styrene acrylate (ASA) as the primary material due to its resistance to degradation [51]. The components that form the enclosure for spectral capture were coated with water-based Musou black paint developed by Koyo Orient Japan Co. (2025), known for its low reflectance index [52]. This treatment minimizes the influence of light reflected by the internal walls of the system. Spectral acquisition is carried out from the Teensy via commands sent through serial communication. This article provides the software developed for the Teensy, along with communication instructions and a graphical user interface tool written in Python, which allows sending the necessary commands to acquire spectral signatures. The full list of commands is included in the section dedicated to operating instructions. The Python software includes buttons for loading and saving acquired spectral signatures in both CSV and image formats. Additionally, the software features a button for executing black calibration (to reduce random noise generated by the system) and another for white calibration (to eliminate the spectral influence of the light source — LED plus incandescent — ensuring that the measured spectrum reflects the properties of the object and not those of the illumination).

• **Low-cost reflectance spectral sensing**: The system enables researchers to capture reflectance spectra in the 340–850 nm range using a compact and affordable setup, which is particularly beneficial for labs with limited budgets or for deployment in remote fieldwork.
• **Modular and customizable design**: Thanks to its 2-inch PVC-based structure and 3D-printed components, the spectrometer can be easily adapted for novel lighting sources, larger samples, or integrated into existing experimental platforms.
• **Integration-ready for IoT and automation systems**: The use of the Teensy 3.2 microcontroller (ARM Cortex-M4) allows straightforward integration with other sensors, actuators, or wireless modules, supporting applications in smart agriculture, environmental monitoring, or automated quality control.
• **Open-source software and interface for rapid deployment**: The included Python-based graphical interface and full command set provide researchers with immediate tools for data acquisition, storage, and calibration, minimizing setup time for custom experiments.
• **Effective for teaching and training**: Due to its low cost, open design, and visible light coverage, the system can be used in educational settings to teach principles of spectroscopy, optical sensing, and hardware–software integration.
• **Soil and material characterization:** The system can be used to analyze the spectral reflectance of soils and construction materials, enabling the estimation of key properties such as moisture, organic matter, and mineral composition. This application supports environmental monitoring, precision agriculture, and quality assessment through a non-destructive and rapid optical approach

## 3 Design files summary

The Design files are summarazed in Table 1.

• **C12880MA SUPPORT:** Structure designed to hold and align the Hamamatsu C12880MA spectrometer.
• **HINGED LID:** Movable cover used to position samples and block external illumination.
• **CHASIS:** Main structural frame that houses and supports all mechanical and electronic components.
• **PVC CASE:** External PVC enclosure that protects the spectrometer and facilitates portability.
• **BOTTOM PANEL:** Plate for providing structural stability and mounting holes for internal components.
• **BASE:** Support element ensuring mechanical balance and proper alignment of the optical assembly.
• **TEENSY BASE:** Mounting platform designed to secure the Teensy microcontroller board.
• **TEENSY COVER:** A cover lid that protects the Teensy board and his connectors.

**Table 1:** Design files of the spectral measurement system.

Design filename	File type	Open source license	Location of the file
C12880MA_SUPPORT	STL	CC-BY-4.0	OSF --- C12880MA_SUPPORT.stl
HINGED_LID	STL	CC-BY-4.0	OSF --- HINGED_LID.stl
CHASIS	STL	CC-BY-4.0	OSF --- CHASIS.stl
PVC_CASE	STL	CC-BY-4.0	OSF --- PVC_CASE.stl
BOTTOM_PANEL	STL	CC-BY-4.0	OSF --- BOTTOM_PANEL.stl
BASE	STL	CC-BY-4.0	OSF --- BASE.stl
TEENSY_BASE	STL	CC-BY-4.0	OSF --- TEENSY_BASE.stl
TEENSY_COVER	STL	CC-BY-4.0	OSF --- TEENSY_COVER.stl
C12880MA_SUPPORT	STEP	CC-BY-4.0	OSF --- C12880MA_SUPPORT.step
HINGED_LID	STEP	CC-BY-4.0	OSF --- HINGED_LID.step
CHASIS	STEP	CC-BY-4.0	OSF --- CHASIS.step
PVC_CASE	STEP	CC-BY-4.0	OSF --- PVC_CASE.step
BOTTOM_PANEL	STEP	CC-BY-4.0	OSF --- BOTTOM_PANEL.step
BASE	STEP	CC-BY-4.0	OSF --- BASE.step
TEENSY_BASE	STEP	CC-BY-4.0	OSF --- TEENSY_BASE.step
TEENSY_COVER	STEP	CC-BY-4.0	OSF --- TEENSY_COVER.step

## 4 Bill of materials summary

This section presents in detail the quantities, prices, and references of the devices and other hardware involved in the system construction process (Table 2).

It is recommended that the M3 Assortment kit include, at a minimum, the following components: two (2) 15 mm countersunk screws, four (4) 6 mm screws, eight (8) 15 mm screws, six (6) lock nuts, and eight (8) standard nuts.

**Table 2:** Bill of materials for the spectral measurement system.

Designator	Component	QTY	Unit-Usd	Total cost-Usd	Source Of materials	Material type
6071	USB-C screw panel	1	$2.50	$2.50	USBC6071	Composite
2756	Teensy 3.2	1	$19.95	$19.95	Teensy 3.2	Composite
C12880-V2	C12880MA sensor	1	$349.99	$349.99	C12880-V2-SENSOR	Composite
482	On/Off switch	1	$4.95	$4.95	On/Off Switch	Composite
1826	MicroB plug	1	$0.95	$0.95	Micro USB Plug	Composite
7207	Incandescent bulb	1	$1.32	$1.32	7207-JKL	Composite
2${}^{\prime \prime }$ pipe	PVC pipe	1	$15.29	$15.29	PVC	Polymer
M3 kit	M3 assortment kit	1	$15.99	$15.99	M3 KIT	Metal
MB-100	Musou black	1	$25.10	$25.10	Musou Black	Composite
LM561C	White led	1	$0.28	$0.28	LED LM561C WARM	Composite
18AWG	Silicon cable	1	$16.09	$16.09	18AWG CABLE	Composite
Resistor	200 ohms resistor	1	$6.05	$0.06	200OHM Resistor	Composite
**Total**				**$442.89**		

## 5 Build instructions

Although the hardware allows for modifications, adjustments, or alternative assembly methods (e.g., omitting the USB Type-C connector, PVC enclosure, or power button), the construction process will be presented as a standardized sequence of steps. Before starting the assembly, it is recommended to have all the 3D-printed mechanical parts prepared. Table 3, Table 4 present the printing parameters, as well as the estimated printing time and the amount of filament required.

As a reference for the construction instructions, Fig. 1(a) shows an exploded view of the system. It is worth noting that this image does not include the electronic components (such as the Teensy board, connectors, or spectral sensor), as their integration is addressed separately in the corresponding sections.

**Table 3:** 3D printing parameters.

Parameter	ASA	PLA
Layer height line width	0.2 mm	0.2 mm
Infill density	50%	100%
Infill pattern	Grid	Grid
Build plate adhesion	Brim	Brim
Printing temperature	260 °C	220 °C
Build plate temperature	100 °C	50 °C
Print speed	200 mm/s	210 mm/s
Generate support	Check	Check
Support structure	Tree	Normal
Support pattern	Grid	Grid
Support density	8%	5%
Regular fan speed	90%	90%

**Table 4:** 3D printing time and filament usage.

	ABS		PLA	
	Printing time	Filament	Printing time	Filament
BASE.step	28 min 31seg	9.50 g	20 min 53seg	9.50 g
BLOCKING_COVER.step	7 min 49seg	3.47 g	8 min 29seg	3.47 g
BOTTOM_PANEL.step	37 min 52seg	17.93 g	27 min 26seg	17.92 g
C12880MA_SUPPORT.step	1 h 15 min	37.46 g	1 h 4 min	37.46 g
CHASIS.step	1 h 18 min	20.56 g	47 min 22seg	20.57 g
HINGED_LID.step	18 min 45seg	12.24 g	17 min 28seg	12.23 g
PVC_CASE.step	2 h 24 min	61.77 g	1 h 42 min	61.77 g
TEENSY_BASE.step	16 min 25seg	8.48 g	15 min 2seg	8.48 g
TEENSY_COVER.step	25 min 27seg	14.10 g	21 min 56seg	14.10 g

1. Parts 1, 3, 5, and 7 contain custom-sized holes designed to hold M3 nuts (nut traps). These nuts should be inserted carefully to avoid damaging the part. To facilitate insertion, a screw can be threaded through the hole and tightened against the properly aligned nut to press it into place.
2. Take parts 2 (HINGED_LID) and 1 (C12880MA_SUPPORT), and apply several uniform coats of Musou Black paint to reduce internal reflectance. It is recommended to apply multiple layers, allowing each coat to dry for at least 20 min before applying the next. In the absence of Musou Black, a low-cost alternative consists of lining the internal chamber with 1000-grit sandpaper dyed with black fabric dye. This approach simulates a low-reflectance optical surface, helping to minimize internal reflections.
3. Take the parts 2 (HINGED_LID) and 1 (C12880MA_SUPPORT) and assemble them as shown in the figure, using a 16 mm M3 screw and a lock nut. Tighten carefully and firmly to avoid damaging the components and to ensure proper hinge operation.
4. Take the BOTTOM_PANEL (5) part and insert the USB Type-C connector and the ON/OFF button. To secure them in place, use the nuts provided by the manufacturer.
5. Connect each of the wires as shown in the schematic diagram 2. If desired, you may change the pin assignments by modifying the corresponding lines in the Teensy code.
6. After completing the wiring, secure the Teensy using the TEENSY_COVER and TEENSY_BASE pieces. To do this, use M3 screws and insert M3 nuts into the corresponding slots to fasten the parts properly.
7. Position the C12880MA sensor on the C12880MA_SUPPORT piece and secure it using the CHASIS and M3 screws. The assembled setup should match the reference shown in the 1(a). Note that the C12880MA_support component has four holes, one of which will be used to mount the 3 mm incandescent light source. (Note: the wires connected to this bulb should be approximately 100 mm long to allow easy handling and connection to the Teensy.)
8. Cut a 15 cm long piece of the PVC pipe. Drill a 3 mm diameter hole at 14 mm from one end, and repeat the process at 17 mm from the opposite end.
9. Insert the BOTTOM_PANEL (5) into one end of the PVC tube. Then take one of the printed BASE pieces and position it on the outside of the tube as shown in Fig. 1(a). Secure it using a countersunk M3 screw.
10. Connect the micro USB cable to the Teensy and insert the assembled structure from step 7 into the PVC tube. Similarly to the previous step, secure the assembly to the tube using countersunk M3 screws.

Fig. 1Spectrometer 3D model and corresponding parts list. (a) 3D model of the spectrometer assembly. (b) Parts List.Fig. 1

**(a):**
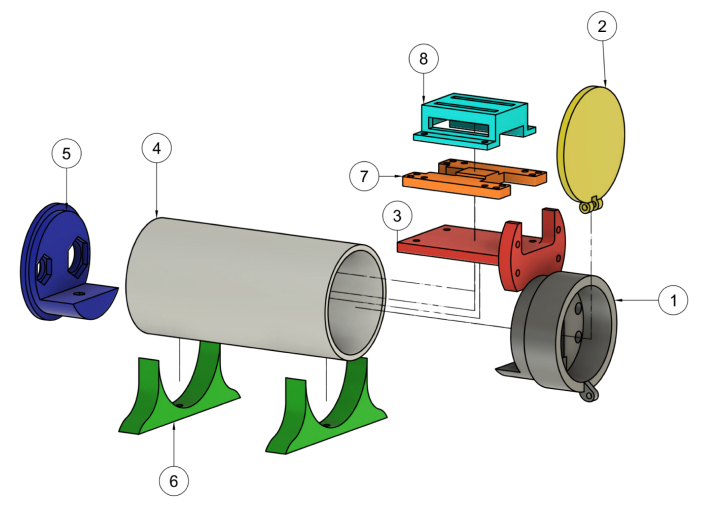

**(b):**
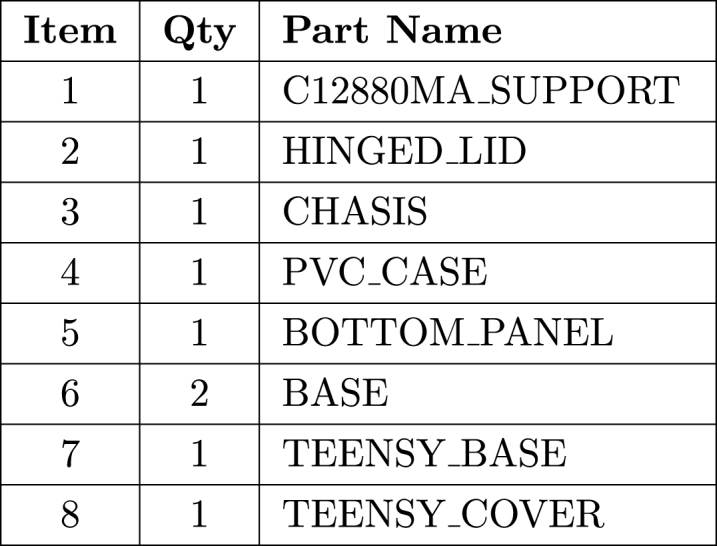

**Fig. 2:**
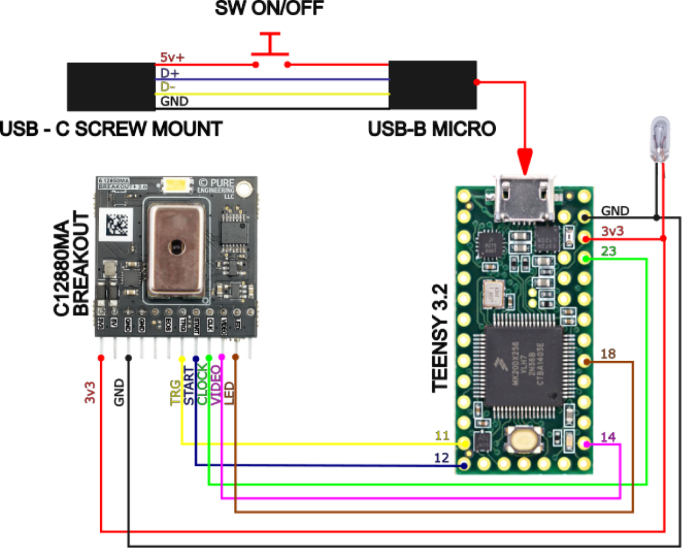
System wiring diagram.

## 6 Operation instructions

Before starting the spectral acquisition process, connect the device to a computer via USB. Ensure that the sample surface is clean, dry, and flat, and that it completely covers the aperture of the capture chamber to avoid ambient light interference. The lid should be fully closed during measurement to maintain a controlled illumination environment. Position the sample so that it remains stable and in full contact with the observation window throughout the acquisition, ensuring accurate and repeatable reflectance measurements. For leaf samples, avoid overlapping or folded areas that could alter the reflectance pattern. After each measurement, remove any residue or dust from the aperture to prevent contamination and maintain optical consistency between acquisitions.

**Fig. 3:**
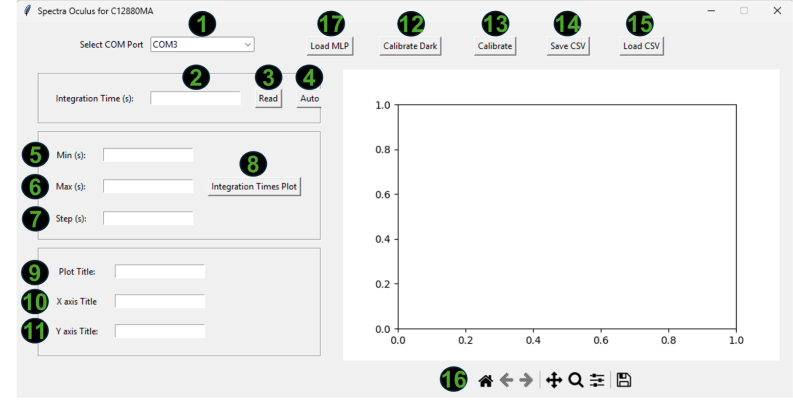
Graphical user interface of the software developed for the reflectance spectrometer based on the Hamamatsu C12880MA sensor. The interface allows the user to select the communication port (1), adjust the integration time (2–4), define acquisition parameters (5–8), customize plot titles (9–11), perform dark and reference calibrations (12–13), and manage data files in CSV format (14–15). At the bottom (16), navigation controls for the plotted spectra are provided. (17) loads the machine learning calibration model.

**Fig. 4:**
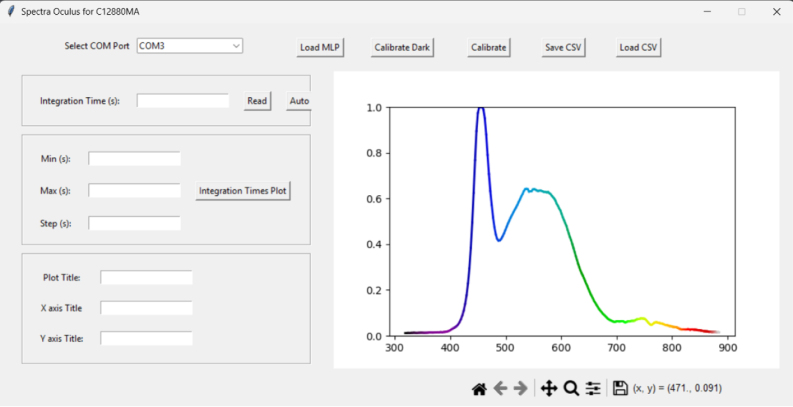
Example of a reflectance spectrum acquired with the developed spectrometer after performing white and dark calibrations. The graph displays the normalized spectral response across the 340–850 nm range, obtained by pressing the **Read** button within the graphical interface.

1. To establish the connection, click on the dropdown list (object 1, Fig. 3) and select the communication port corresponding to the spectrometer. To identify the correct port, it is recommended to check the available ports before connecting the device. After connecting the spectrometer, the corresponding port should appear in the dropdown list.
2. Once the device is selected and the program confirms a successful connection, the calibration process can begin. For this, place the object of interest in front of the spectrometer and click the Auto button in the software. This will allow the system to automatically determine the optimal integration time for capturing the spectral signature. If successful, you should see a screen similar to the one shown in the following figure.
3. After the integration time has been automatically detected, leave the integration time field unchanged and proceed with the dark and white corrections.
4. To perform dark correction, place the black cap on the spectrometer to block all incoming light, and then press the Calibrate Dark button. The signal obtained under these conditions is considered an offset value, as it mainly represents the electronic noise and dark current inherent to the sensor. Consequently, this contribution should not be interpreted as spectral information and is subtracted from the measurements in order to improve radiometric accuracy. If the process is successful, a confirmation message will be displayed.
5. To verify that the dark correction was correctly applied, you can take a new reading with the black cap still in place. The resulting values should be close to zero.
6. After completing the dark correction, proceed with the white correction. Performing both corrections is highly recommended to ensure reliable measurements, performing only one may result in inaccurate results.
7. The spectral contribution of the illumination source is corrected through a white reference adjustment procedure, in which Teflon is used as a reference standard due to its approximately uniform spectral response within the wavelength range of interest. This process allows the measurement to be normalized and ensures that the obtained reflectance corresponds exclusively to the evaluated sample. Furthermore, for a previously defined integration time, the reading associated with this reference value represents the maximum response of the system within that dynamic range. In this way, a controlled saturation level is established that prevents sensor overexposure during subsequent measurements performed under the same acquisition conditions. To perform the white correction, remove the black cap and place a standard white reference material (e.g., Teflon or Spectralon) in front of the sensor. Without changing the integration time, press the Calibrate White button. A confirmation message will appear if the process is successful.
8. You can verify the success of the white correction by taking a new reading of the white reference used. The resulting spectral signature should be composed of values close to one.
9. Finally, after performing both white and dark corrections, it is possible to acquire spectral measurements by pressing the Read button, obtaining results similar to those shown in the following Fig. 4.

Special consideration was given to the stability of the internal optical coatings under varying environmental conditions. The interior surfaces of the measurement chamber are coated with a high-absorption matte paint to minimize internal reflections and improve measurement accuracy. To preserve the optical properties of this coating, the system should be stored and operated in environments with controlled humidity whenever possible. Prolonged exposure to high humidity may degrade the coating over time or promote biological growth such as fungi, which could affect the optical performance of the chamber. For this reason, the device enclosure was designed to reduce environmental exposure, and it is recommended that the instrument be stored in a dry environment and periodically inspected to ensure that the internal surfaces remain clean and free of contamination. These precautions help maintain the long-term stability of the optical characteristics of the measurement chamber.

## 7 Validation and characterization

To ensure the correct operation of the developed spectrometer, a series of tests were conducted to validate its performance in both radiometry and reflectance spectrometry.

### 7.1 Radiometry validation

The first set of experiments evaluated the device in spectroradiometer mode. Measurements were performed using three different light sources (blue laser, red laser, and ambient white LED illumination) and were compared with the spectral signatures obtained from the commercial reference spectrometer HOPOO OHSP-350 (hereinafter referred to as OHSP). The OHSP spectrometer has a spectral resolution of 0.2 nm and an operating range between 380 and 780 nm. Although this range is narrower than that of the developed system (340–840 nm), there is an overlap of approximately 80%, which allows for a direct comparison across most of the measured region. To validate wavelength detection accuracy, two laser beams with nominal emissions at 405 nm (blue) and 650 nm (red) were used. Additional tests included measurements of ambient light, the prototype’s integrated illumination systems (a white LED and an incandescent bulb), and sunlight. These measurements enabled assessment of the device’s response across different regions of the spectrum.

The system response under the blue laser exhibited a peak at 407 nm, as shown in Fig. 5(a). This result is consistent with the source emission and the OSHP measurements, with a mean squared error of $6.6325\times 10^{-3}$, corresponding to approximately 8.14%. Similarly, in the red laser analysis (Fig. 5(b)), the system response exhibited a peak at 663 nm, consistent with the source emission and the OSHP measurements, with a mean squared error of $1.4293\times 10^{-2}$, corresponding to approximately 11.9%.

Simultaneous measurements of the ambient light spectrum using both the developed open source system and the OSHP spectrometer showed minimal variability, with consistent signatures across the evaluated wavelengths. Fig. 6(a) presents a comparison between the proposed system and the reference ground truth. The acquired measurements yielded a mean squared error of $6.005\times 10^{-3}$, corresponding to approximately 7.74%.Fig. 5Blue Laser and Red Laser spectral response. (a) Blue laser spectral response. The dotted line represents the OSHP measurements, whereas the solid blue line represents the measurements obtained with open source system. (b) Red Lase spectral Response. The dotted line represents the OSHP measurements, whereas the solid blue line represents the measurements obtained with the open source system.Fig. 5

**(a):**
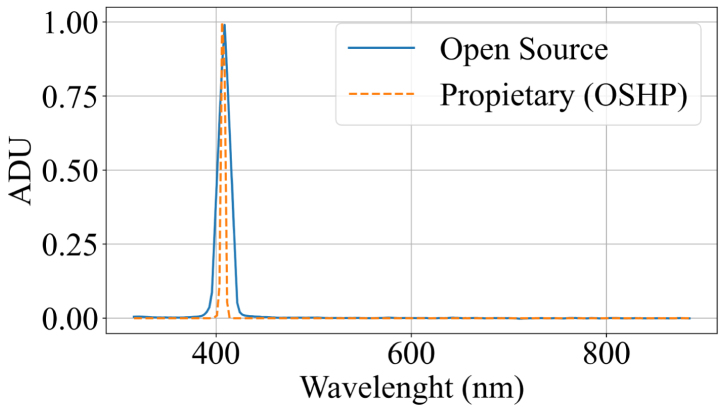

**(b):**
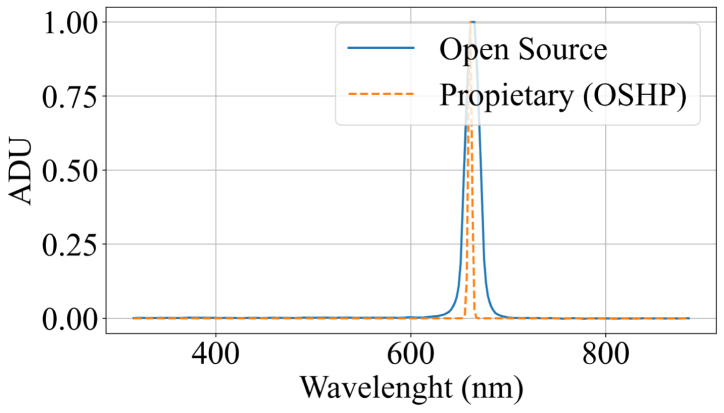

The developed system exhibited a bell-shaped response curve, deviating from the expected incandescent profile, which should display a monotonic increase in the infrared region. In contrast, the reference OHSP measurements confirmed this behavior, yielding a mean squared error (MSE) of $1.029\times 10^{-1}$, corresponding to approximately 32%. Beyond 700 nm, the acquired signature diverged from the theoretical trend, which can be attributed to the reduced sensitivity of the sensor below 400 nm and above 780 nm, as reported in [53]. This behavior is also observed in the spectral response of the incandescent bulb, which begins to decrease around 780 nm 6(b) . Consequently, the spectral extremes tend to saturate and distort the signal. To mitigate this effect, the use of higher-power illumination or a region-specific acquisition strategy, employing different light sources tailored to the spectral zone of interest, is recommended.Fig. 6White LED ambient illumination and incandescent bulb spectral response. (a) White LED ambient illumination spectral response. The dotted line represents the OSHP measurements, whereas the solid blue line represents the measurements obtained with the open source system. (b) Incandescent bulb spectral response. The dotted line represents the OSHP measurements, whereas the solid blue line represents the measurements obtained with the open source system.Fig. 6

**(a):**
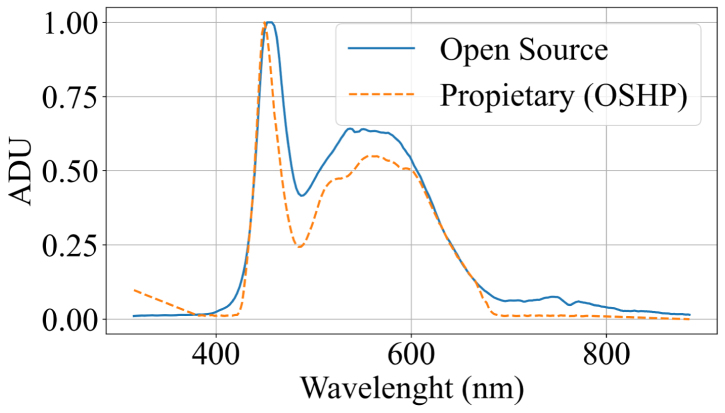

**(b):**
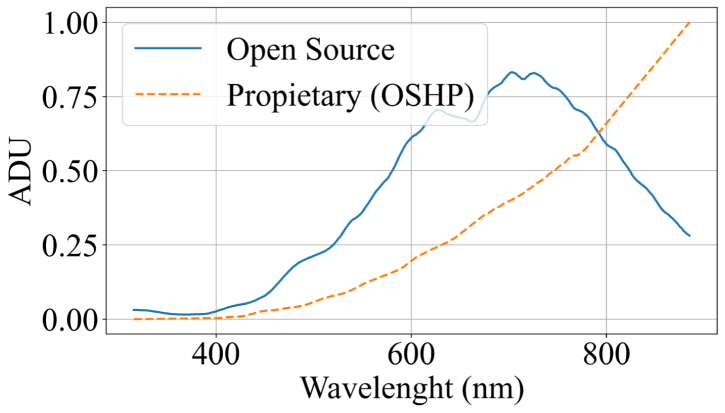

### 7.2 Reflectance validation

Reflectance validation was conducted using a ColorChecker Classic [54], a widely employed tool in color calibration. This standard includes 24 patches covering natural, chromatic, primary, and grayscale tones, designed under scientific criteria and extensively validated in spectrometry studies.

As a reference, measurements acquired using the CI-710s SpectraVue Leaf Spectrometer were employed. This instrument operates over a spectral range of 360–1100 nm with a full width at half maximum (FWHM) of 2.4 nm, enabling a direct comparison with the developed system.

Due to the limited emission of the illumination sources in the proposed system below 400 nm, a noticeable discrepancy is observed in this region across the spectra obtained from the ColorChecker samples. The measurements obtained with the developed system were consistent with the reference data with an MSE = 0.0159 and a RMSE = 12.63%, confirming its suitability for material identification and characterization. Table 5 presents the MSE and RMSE of the system for each spectral response of the 24 patches, with respect to the reference data.

Given that the illumination sources used do not exhibit a perfectly uniform spectral distribution over the wavelength range covered by the sensor, a calibration process based on a multilayer perceptron neural network is implemented. This model enables the compensation of spectral variations inherent to the illumination source and the optical system, mitigating systematic distortions and improving the quality, consistency, and reliability of the acquired measurements. The model was configured with three hidden layers containing 36, 20, and 10 neurons, respectively. The *tanh* activation function and the *L-BFGS* optimizer were employed, the latter being suitable for regression tasks with moderately sized datasets. The initial learning rate was set to 0.01, with a maximum of 5000 iterations and a fixed random seed of 42 to ensure reproducibility. Input features were previously normalized, and the training process incorporated both original data and synthetic samples generated through linear combinations of input vectors and their corresponding reference values, in order to enhance the model’s stability and generalization capability. The results obtained from the system calibration process show a mean squared error (MSE) of 0.0028 and a root mean squared error (RMSE) of 5.35%. The results for each patch are presented in Table 6, highlighting the adjustment values achieved by the model under the evaluated conditions. The training process is available at the following Colab Notebook.

**Table 5:**
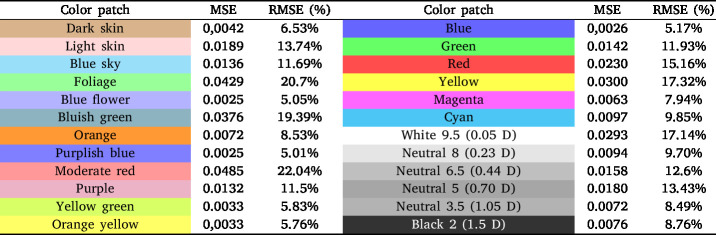
MSE and RMSE of the spectral responses for the 24 patches.

Additionally, in order to validate the system performance on real samples, spectral signatures from a green and a yellow leaf of *Epipremnum aureum* were acquired and compared with those obtained using the SpectraVue instrument. The samples under study are shown in Fig. 7.

**Table 6:**
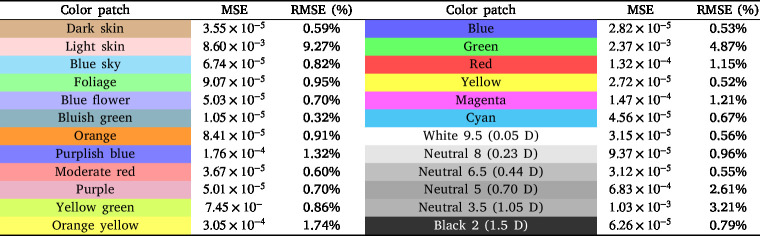
MLP calibration results (MSE and RMSE) for each patch.

The results are presented in Fig. 8 using the previously trained model. These results demonstrate that the model is capable of applying the necessary corrections in spectral bands affected by insufficient illumination, thereby confirming the system’s capability to acquire reflectance-based spectral measurements.Fig. 7Green and Yellow Epipremnum Aureum leaf samples. (a) Green Epipremnum Aureum Leaf. (b) Yellow Epipremnum Aureum Leaf.Fig. 7

**(a):**
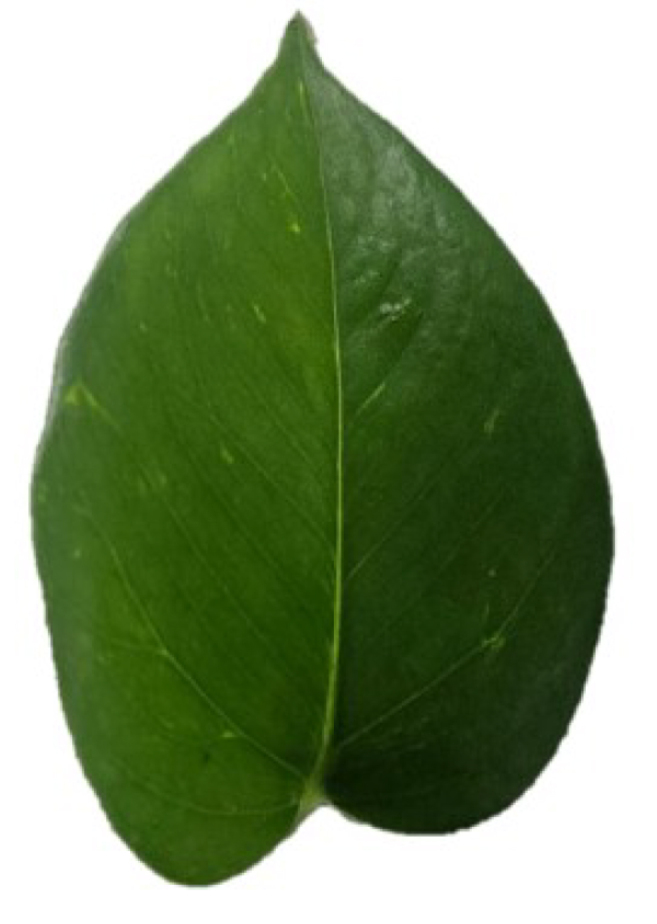

**(b):**
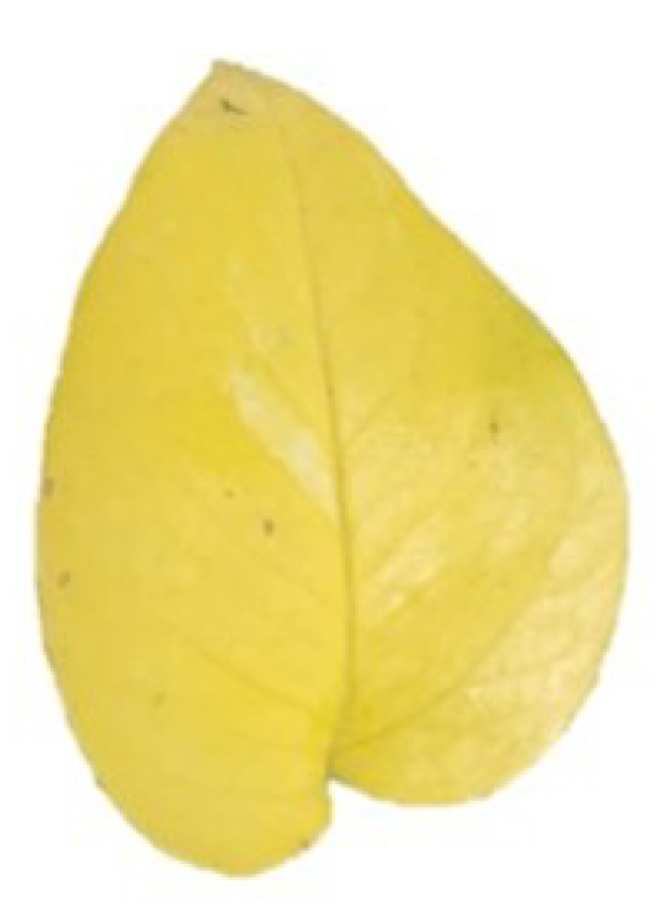

Fig. 8Green and Yellow Epipremnum Aureum spectral signatures. (a) Green Epipremnum Aureum Leaf spectral signature. MSE = 0.0084 and RMSE = 9.2%. (b) Yellow Epipremnum Aureum Leaf spectral signature. MSE = 0.0391 and RMSE = 19.7%.Fig. 8

**(a):**
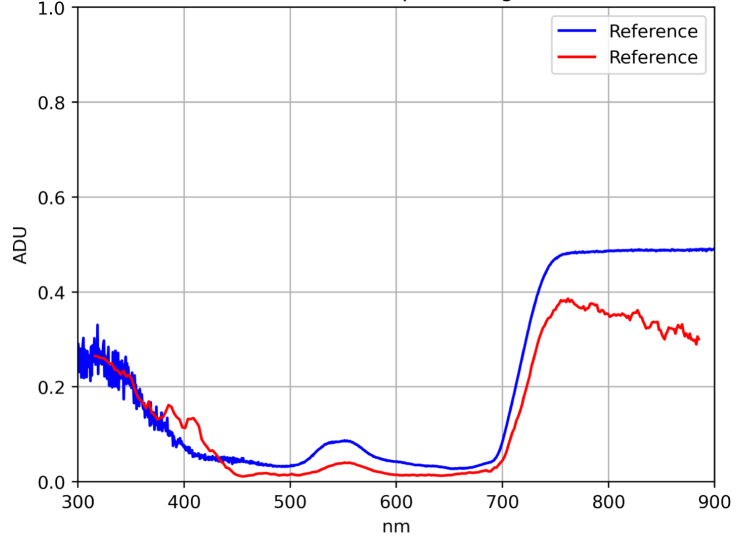

**(b):**
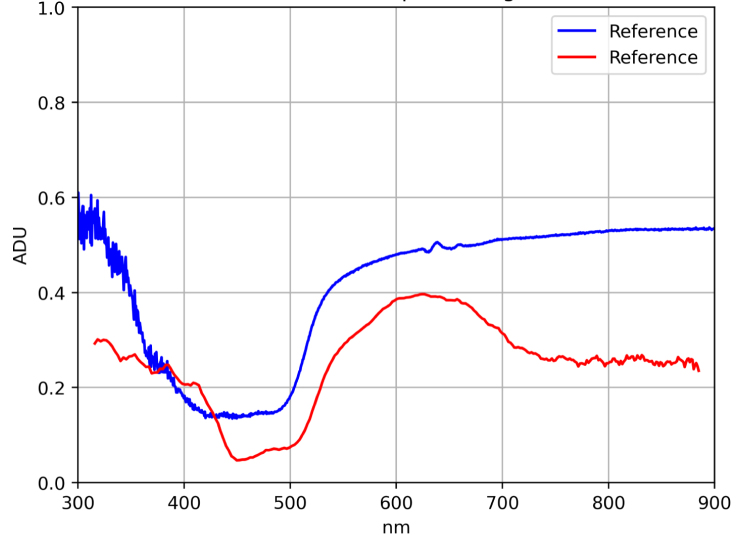

### 7.3 Conclusions and future work

This article has demonstrated a low-cost, open-source spectrometer with a total system cost of approximately $442.89. Its design leverages digitally distributed 3D-printed components and easily sourced parts, ensuring high replicability for educational and research purposes. The system provides direct access to raw spectral data, enabling the flexible calculation of a wide range of user-defined spectral indices for specific applications. It successfully characterizes material reflectance and light source radiometry, validated using OHSP-350 spectrometer and SpectraVue leaf spectrometer. The device captures spectral data from 340 nm to 840 nm, making it a practical tool for applications in agriculture and material science. Future work can further develop the device. Development is underway to integrate an onboard battery and display, creating a fully standalone field-deployable unit for streamlined data capture. The system requires calibration using a Multilayer Perceptron model to achieve high accuracy. Uncalibrated measurements can show errors of up to 12.63%, which are significantly reduced post-calibration.

### 7.4 Prototype functionality and limitations

#### 7.4.1 Functionality/capabilities:

• Captures spectral data from 340 nm to 840 nm.
• Provides direct access to raw spectral data for flexible calculation of user-defined spectral indices.
• Successfully characterizes material reflectance and light source radiometry.
• Low-cost and open-source, with high replicability using 3D-printed components and readily available parts.
• Validated against commercial spectrometers ({OHSP-350 and SpectraVue leaf spectrometer).
• Suitable for educational and research applications in agriculture, material science, and other domains.
• Future upgrades planned: onboard battery and display for fully standalone, field-deployable use.

#### 7.4.2 Limitations/considerations

• High measurement accuracy requires calibration using an MLP model; uncalibrated measurements can have errors up to 12.63%.
• Optical performance depends on correct assembly and proper treatment of internal coatings (e.g., Musou Black or low-cost alternatives).
• Sensor and illumination limitations reduce spectral accuracy at wavelength extremes (<400nm and >700nm).
• Environmental factors such as humidity and temperature may affect long-term stability of coatings and optical components.
• Field deployment may require careful handling to preserve calibration and optical performance.

## CRediT authorship contribution statement

**M. Mejia-Herrera:** Writing – review & editing. **J.S. Botero-Valencia:** Writing – review & editing. **J.F. Vargas-Bonilla:** Writing – review & editing. **J.M. Pearce:** Writing – review & editing.

## Declaration of competing interest

The authors declare that they have no known competing financial interests or personal relationships that could have appeared to influence the work reported in this paper.
